# A HS-PRP-Type Hybrid Conjugate Gradient Method with Sufficient Descent Property

**DOI:** 10.1155/2021/2087438

**Published:** 2021-10-22

**Authors:** Xiaodi Wu, Yihan Zhu, Jianghua Yin

**Affiliations:** ^1^College of Mathematics and Physics, Guangxi University for Nationalities, Nanning 530006, China; ^2^College of Mathematics and Computer Science, Guangxi Science and Technology Normal University, Laibin 546199, China

## Abstract

In this paper, based on the HS method and a modified version of the PRP method, a hybrid conjugate gradient (CG) method is proposed for solving large-scale unconstrained optimization problems. The CG parameter generated by the method is always nonnegative. Moreover, the search direction possesses the sufficient descent property independent of line search. Utilizing the standard Wolfe–Powell line search rule to yield the stepsize, the global convergence of the proposed method is shown under the common assumptions. Finally, numerical results show that the proposed method is promising compared with two existing methods.

## 1. Introduction

Consider the problem of minimizing *f* over *R*^*n*^:

(1) $$\begin{array}{c} \underset{x\in R^{n}}{\min}\;f\left(x\right), \end{array}$$

where *f* : *R*^*n*^⟶*R* is continuously differentiable. Throughout, the gradient of *f* at *x* is denoted by *g*(*x*), i.e., *g*(*x*) : =∇*f*(*x*). We know that conjugate gradient (CG) methods are very popular and effective for solving unconstrained optimization problems (1), especially for large-scale case by means of their simplicity and low memory requirements. These preferred features greatly promote their applications in various areas such as image deblurring and denoising, neural network, compressed sensing, and others. We refer the interested readers to some recent works [1–3] and references therein for more details. The numerical results reported in [1] reveal that the CG method has great potential in solving image restoration problems.

Generally, the iterative formula of the CG method for solving problem (1) can be read as

(2) $$\begin{array}{c} x_{k+1}=x_{k}+\alpha_{k}d_{k}, \end{array}$$

where *α*_*k*_ > 0 is called the stepsize computed by some line search. Here, *d*_*k*_ is commonly known as the search direction, which is defined as follows:

(3) $$\begin{array}{c} d_{k}=\left\{\begin{array}{cc} -g_{k}, & \text{if }k=1,\\ -g_{k}+\beta_{k}d_{k-1}, & \text{if }k\geq 2, \end{array}\right) \end{array}$$

where *β*_*k*_ ∈ *R* is the so-called CG parameter and *g*_*k*_ is the abbreviation of *g*(*x*_*k*_), i.e., *g*_*k*_ : =*g*(*x*_*k*_). The two key factors that affect the numerical performance of the CG method are the stepsize and the CG parameter. First, we outline several well-known line search criteria in the literature.

(a) The exact line search rule: calculate a stepsize *α*_*k*_ satisfying
  (4) $$\begin{array}{c} f\left(x_{k}+\alpha_{k}d_{k}\right)=\underset{\alpha \geq 0}{\min}f\left(x_{k}+\alpha d_{k}\right). \end{array}$$
(b) The standard (weak) Wolfe–Powell (WWP) line search rule: calculate a stepsize *α*_*k*_ satisfying
  (5) $$\begin{array}{c} f\left(x_{k}+\alpha_{k}d_{k}\right)\leq f\left(x_{k}\right)+\delta \alpha_{k}g_{k}^{T}d_{k}, \end{array}$$
and
  (6) $$\begin{array}{c} g{\left(x_{k}+\alpha_{k}d_{k}\right)}^{T}d_{k}\geq \sigma g_{k}^{T}d_{k}, \end{array}$$
where 0 < *δ* < *σ* < 1.
(c) The strong Wolfe–Powell (SWP) line search rule: calculate a stepsize *α*_*k*_ satisfying (5) and
  (7) $$\begin{array}{c} \left|g{\left(x_{k}+\alpha_{k}d_{k}\right)}^{T}d_{k}\right|\leq \sigma \left|g_{k}^{T}d_{k}\right|. \end{array}$$

On the other hand, different CG methods are determined by different CG parameters. The well-known CG methods include the Fletcher–Reeves (FR) [4], Polak–Ribière–Polyak (PRP) [5, 6], Hestenes–Stiefel (HS) [7], Liu–Storey (LS) [8], Fletcher (CD) [9], and Dai–Yuan (DY) [10] methods, and their CG parameters *β*_*k*_ are, respectively, given by

(8) $$\begin{array}{c} \beta_{k}^{\text{FR}}=\frac{{\left\|g_{k}\right\|}^{2}}{{\left\|g_{k-1}\right\|}^{2}},\\ \beta_{k}^{\text{PRP}}=\frac{g_{k}^{T}y_{k-1}}{{\left\|g_{k-1}\right\|}^{2}},\\ \beta_{k}^{\text{HS}}=\frac{g_{k}^{T}y_{k-1}}{d_{k-1}^{T}y_{k-1}},\\ \beta_{k}^{\text{LS}}=-\frac{g_{k}^{T}y_{k-1}}{g_{k-1}^{T}d_{k-1}},\\ \beta_{k}^{\text{CD}}=-\frac{{\left\|g_{k}\right\|}^{2}}{d_{k-1}^{T}g_{k-1}},\\ \beta_{k}^{\text{DY}}=\frac{{\left\|g_{k}\right\|}^{2}}{d_{k-1}^{T}y_{k-1}}, \end{array}$$

where *y*_*k*−1_ : =*g*_*k*_ − *g*_*k*−1_ and ‖·‖ stands for the Euclidean norm. The methods yielded by the above CG parameters are called the classical CG methods, and their convergence analysis and numerical performance have been extensively studied (see, e.g., [4–12]). It has been shown that the above formulas for the CG parameters are equivalent when *f*(*x*) is convex quadratic and the stepsize *α*_*k*_ is obtained by carrying out the exact line search rule (4). However, their numerical performance strongly depends on the CG parameter *β*_*k*_. The FR, CD, and DY methods possess good convergence, but the numerical performance for these methods is somewhat unsatisfactory for solving general unconstrained nonlinear optimization problems [12–14]. On the contrary, it has been shown that the convergence properties of PRP, HS, and LS methods are not so well, but they often possess better computational performance [12–14]. Therefore, in the past few decades, based on the above formulas, plenty of formulas for *β*_*k*_ are designed for CG methods that possess both good global convergence properties and promising numerical performance (see [12–16] and references therein).

To our knowledge, the first hybrid CG method in the literature was proposed by Touati-Ahmed and Storey [17] (TS method), where *β*_*k*_ is computed as

(9) $$\begin{array}{c} \beta_{k}^{\text{TS}}=\left\{\begin{array}{cc} \beta_{k}^{\text{PRP}}, & \text{if }0\leq \beta_{k}^{\text{PRP}}\leq \beta_{k}^{\text{FR}},\\ \beta_{k}^{\text{FR}}, & \text{otherwise}. \end{array}\right) \end{array}$$

Apparently, the TS method has some good properties of FR and PRP methods since *β*_*k*_^TS^ is a hybrid of *β*_*k*_^FR^ and *β*_*k*_^PRP^. Combined with HS and DY methods, Dai and Yuan [18] proposed another hybrid CG method (hHD method), in which the hybrid CG parameter *β*_*k*_ is obtained by

(10) $$\begin{array}{c} \beta_{k}^{\text{hHD}}=\max\left\{0,\min\left\{\beta_{k}^{\text{HS}},\beta_{k}^{\text{DY}}\right\}\right\}. \end{array}$$

When the WWP line search rule is used to compute the stepsize, the resulting search direction in [18] is a descent one and the global convergence for the hHD method is proved. Moreover, the numerical experiments reported in [18] illustrated that the hHD method is competitive and practicable. For other closely related works, we refer the readers to [18, 19] and the references therein. It is worth noting that the CG parameters *β*_*k*_ defined in [17–19] are restricted to positive values. As explicated in [19], this restriction in turn results in global convergence of the algorithm. In recent years, many hybrid CG methods were proposed on the basis of the methodology of discrete combinations of several CG parameters (see, e.g., [1, 13, 20–23]). The combination parameter is computed by some secant equations [13, 20], the conjugacy condition [21, 22], or by minimizing the least-squares problem consisting of the unknown search direction and an existing one (see [23] and the references therein).

In 2016, Wei et al. [24] introduced a modified PRP method, usually called the WYL method, where the corresponding parameter *β*_*k*_ is yielded by

(11) $$\begin{array}{c} \beta_{k}^{\text{WYL}}=\frac{{\left\|g_{k}\right\|}^{2}-\left\|g_{k}\right\|/\left\|g_{k-1}\right\|g_{k}^{T}g_{k-1}}{{\left\|g_{k-1}\right\|}^{2}}. \end{array}$$

Under the assumption that *d*_*k*_ generated by Wei et al. [24] satisfies the so-called sufficient descent condition

(12) $$\begin{array}{c} g_{k}^{T}d_{k}\leq -c{\left\|g_{k}\right\|}^{2},\;c>0, \end{array}$$

the WYL method is globally convergent under the WWP line search rule and possesses superior numerical performance. Subsequently, Dai and Wen [25] proposed two improved CG methods with sufficient descent property. The CG parameters *β*_*k*_ in [25] are defined as

(13) $$\begin{array}{c} \beta_{k}^{\text{DHS}}=\frac{{\left\|g_{k}\right\|}^{2}-\left\|g_{k}\right\|/\left\|g_{k-1}\right\|\left|g_{k}^{T}g_{k-1}\right|}{d_{k-1}^{T}y_{k-1}+\mu \left|g_{k}^{T}d_{k-1}\right|},\\ \beta_{k}^{\text{DPRP}}=\frac{{\left\|g_{k}\right\|}^{2}-\left\|\left|g_{k}\right|\right\|/\left\|g_{k-1}\right\|\left|g_{k}^{T}g_{k-1}\right|}{{\left\|g_{k-1}\right\|}^{2}+\mu \left|g_{k}^{T}d_{k-1}\right|}, \end{array}$$

where *μ* > 1. Clearly, the search direction yielded by *β*_*k*_^DPRP^ satisfies the sufficient descent condition without depending on any line search. However, the sufficient descent property associated with *β*_*k*_^DHS^ relies on the WWP line search rule.

Based on the above observations, it is interesting to design a hybrid CG method such that the CG parameter is nonnegative and the resulting search direction possesses the sufficient descent property independent of line search technique. Motivated by the methods in [24, 25] and considering that the HS method performs best among the classical CG methods, a new formula for the CG parameter *β*_*k*_ is given by

(14) $$\begin{array}{c} \beta_{k}^{\text{hHPR}}=\min\left\{\left|\beta_{k}^{\text{HS}}\right|,\frac{{\left\|g_{k}\right\|}^{2}-\left\|g_{k}\right\|/\left\|g_{k-1}\right\|g_{k}^{T}g_{k-1}}{{\left\|g_{k-1}\right\|}^{2}+\gamma \left|g_{k}^{T}d_{k-1}\right|}\right\}, \end{array}$$

where *γ* > 2. It is not difficult to see that *β*_*k*_^hHPR^ is a hybrid of *β*_*k*_^HS^, *β*_*k*_^WYL^, and *β*_*k*_^DPRP^. Interestingly, the above parameter *β*_*k*_^hHPR^ is always nonnegative. To see this, let *θ*_*k*_ be the angle between *g*_*k*_ and *g*_*k*−1_. Thus, we know from (14) that

(15) $$\begin{array}{c} \beta_{k}^{\text{hHPR}}\leq \frac{{\left\|g_{k}\right\|}^{2}-\left\|g_{k}\right\|/\left\|g_{k-1}\right\|g_{k}^{T}g_{k-1}}{{\left\|g_{k-1}\right\|}^{2}+\gamma \left|g_{k}^{T}d_{k-1}\right|}=\frac{{\left\|g_{k}\right\|}^{2}\left(1-\cos\;\theta_{k}\right)}{{\left\|g_{k-1}\right\|}^{2}+\gamma \left|g_{k}^{T}d_{k-1}\right|}\leq \frac{2{\left\|g_{k}\right\|}^{2}}{{\left\|g_{k-1}\right\|}^{2}+\gamma \left|g_{k}^{T}d_{k-1}\right|}, \end{array}$$

which further implies

(16) $$\begin{array}{c} 0\leq \beta_{k}^{\text{hHPR}}\leq \frac{2{\left\|g_{k}\right\|}^{2}}{{\left\|g_{k-1}\right\|}^{2}}. \end{array}$$

Moreover, plugging the CG parameter *β*_*k*_ : =*β*_*k*_^hHPR^ into (3), we can show that the resulting search direction possesses the sufficient descent property independent of line search technique (see Lemma 1 below).

The structure of this paper is organized as follows. In Section 2, our algorithm framework is presented, and the sufficient descent property with respect to the resulting search direction is discussed in detail.  Section 3 is devoted to establishing the convergence of the proposed method with the WWP line search rule. In the last section, some preliminary numerical results are reported to verify the efficiency of the presented method.

## 2. The Algorithm

In this section, we first propose the algorithm framework for solving problem (1), in which we do not specify which line search rule generates the stepsize. Subsequently, we analyze the sufficient descent property for the search direction. By inserting the WWP line search rule into the algorithm framework, our hybrid CG method is proposed.

The following lemma shows that the direction sequence {*d*_*k*_} generated by Algorithm 1 possesses the sufficient descent property independent of any line search.

**Lemma 1 .**

Let {*d*_*k*_} be a sequence generated by Algorithm 1. Then, for some constant *M* ∈ (0,1), it holds that

(17) $$\begin{array}{c} g_{k}^{T}d_{k}\leq -M{\left\|g_{k}\right\|}^{2},\;\forall ,k\geq 1. \end{array}$$

**Proof**

When *k*=1, it follows from the definition of *d*_*k*_ in (3) that *g*_1_^*T*^*d*_1_=−‖*g*_1_‖^2^ ≤ −*M*‖*g*_1_‖^2^. So, the relation in (17) holds when *k*=1. Now, consider the case *k* ≥ 2. If *g*_*k*_^*T*^*d*_*k*−1_=0, it follows from (3) that

(18) $$\begin{array}{c} g_{k}^{T}d_{k}=-{\left\|g_{k}\right\|}^{2}\leq -M{\left\|g_{k}\right\|}^{2}. \end{array}$$

Suppose that *g*_*k*_^*T*^*d*_*k*−1_ ≠ 0 for all *k* ≥ 2. It then follows from (3), (15), and (16) that

(19) $$\begin{array}{c} g_{k}^{T}d_{k}=-{\left\|g_{k}\right\|}^{2}+\beta_{k}^{\text{hHPR}}g_{k}^{T}d_{k-1}\\ \leq -{\left\|g_{k}\right\|}^{2}+\frac{2{\left\|g_{k}\right\|}^{2}}{{\left\|g_{k-1}\right\|}^{2}+\gamma \left|g_{k}^{T}d_{k-1}\right|}\left|g_{k}^{T}d_{k-1}\right|\\ \leq -{\left\|g_{k}\right\|}^{2}+\frac{2{\left\|g_{k}\right\|}^{2}}{\gamma \left|g_{k}^{T}d_{k-1}\right|}\left|g_{k}^{T}d_{k-1}\right|\\ \leq -\left(1-\frac{2}{\gamma }\right){\left\|g_{k}\right\|}^{2}≕-M{\left\|g_{k}\right\|}^{2}, \end{array}$$

which completes the proof.

For convenience, in the following statements, we call the method generated by Algorithm 1 with the WWP line search rule as the hHPR CG method.

## 3. Convergence

In this section, we analyze the convergence for the hHPR CG method. For this goal, the following common assumptions are necessary.

**Assumption 1 .**

(i) The level set Ω={*x* ∈ *R*^*n*^*|f*(*x*) ≤ *f*(*x*_1_)} is bounded. Here, *x*_1_ is the given initial point.
(ii) In some neighborhood *N* of the level set Ω, the objective function *f*(*x*) is continuously differentiable, and its gradient *g*(*x*) is Lipschitz continuous, i.e., there exists a constant *L* > 0 such that
  (20) $$\begin{array}{c} \left\|g\left(x\right)-g\left(y\right)\right\|\leq L\left\|x-y\right\|,\;\forall x,y\in N. \end{array}$$

The following lemma provides the convergence for the PRP-type CG method, which was originally introduced in [19].

**Lemma 2 .**

Consider the general CG method (2) and (3) with the following three properties:

(i) The CG parameter is always nonnegative, i.e., *β*_*k*_ ≥ 0 for all *k* ≥ 1.
(ii) The line search satisfies (5) and (6) and the sufficient descent condition.
(iii) Property (*∗*) holds. Then,
  (21) $$\begin{array}{c} \underset{k⟶\infty }{\lim\inf}\left\|g_{k}\right\|=0. \end{array}$$

**Property 1 .**

(*∗*) Consider a method of forms (2) and (3). Suppose that

(22) $$\begin{array}{c} 0<\gamma \leq \left\|g_{k}\right\|\leq \bar{\gamma },\;\forall ,k\geq 1. \end{array}$$

We say that the method has property (*∗*), if for all *k* ≥ 1, there exist constants *b* > 1 and *λ* > 0 such that |*β*_*k*_| ≤ *b*, and if ‖*s*_*k*−1_‖ ≤ *λ* where *s*_*k*−1_=*x*_*k*_ − *x*_*k*−1_, then we have |*β*_*k*_| ≤ 1/2*b*.

From (16) and Lemmas 1 and 2, to obtain the global convergence of the hHPR CG method, we only prove that our method owns property (*∗*).

**Lemma 3 .**

Consider the method of forms (2) and (3) in which *β*_*k*_=*β*_*k*_^hHPR^. If Assumption 1 holds, then *β*_*k*_^hHPR^ satisfies property (*∗*).

**Proof**

Considering the method of forms (2) and (3) and using the constants *γ* and $\bar{\gamma }$ in (22), we have from (16) that

(23) $$\begin{array}{c} 0\leq \beta_{k}^{\text{hHPR}}\leq \frac{2{\left\|g_{k}\right\|}^{2}}{{\left\|g_{k-1}\right\|}^{2}}\leq \frac{2{\bar{\gamma }}^{2}}{\gamma^{2}}. \end{array}$$

Let $b=2{\bar{\gamma }}^{2}/\gamma^{2}\geq 2$ and $\lambda =\gamma^{4}/8L{\bar{\gamma }}^{3}>0$. If ‖*s*_*k*−1_‖ ≤ *λ*, we obtain from Assumption 1(ii) and (15) that

(24) $$\begin{array}{c} \beta_{k}^{\text{hHPR}}\leq \frac{g_{k}^{T}\left(g_{k}-\left\|g_{k}\right\|/\left\|g_{k-1}\right\|g_{k-1}\right)}{{\left\|g_{k-1}\right\|}^{2}}\\ \leq \frac{\left\|g_{k}\right\|\cdot \left\|g_{k}-g_{k-1}+g_{k-1}-\left\|g_{k}\right\|/\left\|g_{k-1}\right\|g_{k-1}\right\|}{{\left\|g_{k-1}\right\|}^{2}}\\ \leq \frac{\left\|g_{k}\right\|\cdot \left\|g_{k}-g_{k-1}\right\|+\left\|g_{k}\right\|\cdot \left|\left\|g_{k-1}\right\|-\left\|g_{k}\right\|\right|}{{\left\|g_{k-1}\right\|}^{2}}\\ \leq \frac{2\left\|g_{k}\right\|\left\|g_{k}-g_{k-1}\right\|}{{\left\|g_{k-1}\right\|}^{2}}\leq \frac{2L\left\|s_{k-1}\right\|\left\|g_{k}\right\|}{{\left\|g_{k-1}\right\|}^{2}}\\ \leq \frac{2L\lambda \bar{\gamma }}{\gamma^{2}}=\frac{1}{2b}. \end{array}$$

Therefore, the proof is completed.

With (16) and Lemmas 1–3 at hand, one can establish the global convergence of the hHPR CG method.

**Theorem 1 .**

Let {*x*_*k*_} be a sequence generated by the hHPR CG method. If Assumption 1 holds, then lim inf_*k*⟶*∞*_‖*g*_*k*_‖=0.

## 4. Numerical Experiments

In this section, we verify the efficiency and robustness of the hHPR CG method (hHPR for short) by solving some classical tested problems and compare it with two well-known CG methods: DHS and DPRP in [25].

For the tested problems, some of them are from the well-known CUTE library in [26] and the others come from [27]. Moreover, their dimensions range from 2 to 1000000. All codes were written in MATLAB R2016a, and the numerical experiments were conducted on a Dell PC with Intel Core CPU 3.00 GHz and 16.00 GB RAM. For the aforementioned methods, we reset the search direction by taking *d*_*k*_ : =−*g*_*k*_ once an ascent direction occurs. For the sake of fairness, all the stepsizes *α*_*k*_ are yielded by the WWP line search rule following a bisection algorithm proposed in [28], and the corresponding parameters are set to *δ*=0.01 and *σ*=0.1. Moreover, we adopt the strategy described in [29] to compute the initial stepsize.

Let *γ*=3 for hHPR, and let *μ*=2 for DHS and DPRP. Denote the iteration numbers, the CPU time in seconds, and the final value of ‖*g*_*k*_‖ by Itr,  Tcpu, and ‖*g*_*∗*_‖, respectively. If ‖*g*_*k*_‖ ≤ 10^−6^ or Itr > 2000, we stop the program. If the latter requirement holds, i.e., Itr > 2000, we use “-” to denote Itr, Tcpu, and ‖*g*_*∗*_‖.

The numerical results are listed in Tables 1 and 2, where “TP” denotes the tested problems used in numerical experiments and “Dim” stands for the dimension of the tested problems.

**Figure 1:**
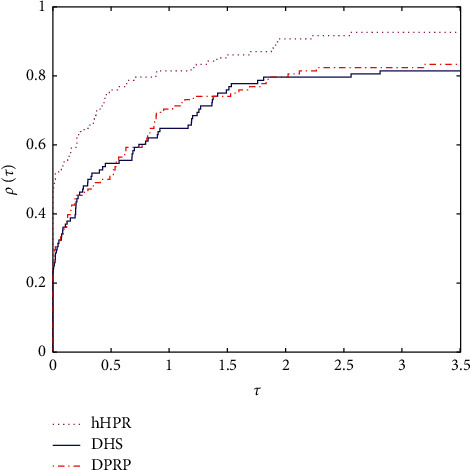
Performance profile on Tcpu.

**Figure 2:**
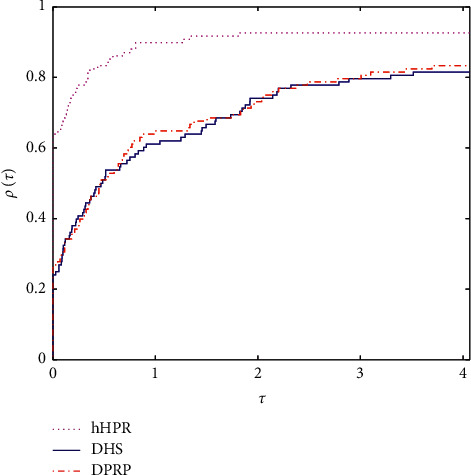
Performance profile on Itr.

As we all know, the performance profile introduced in [30] is very useful in measuring the performance of numerical algorithms. Figures 1 and 2 plot the performance profiles of hHPR, DHS, and DPRP in terms of Itr and Tcpu, respectively. Based on the left side of Figures 1 and 2, the proposed method is clearly above the other two curves, and this in turn shows that compared with DHS and DPRP, our proposed method is efficient and encouraging. On the other hand, based on the right side of Figures 1 and 2, our proposed method can successfully solve about 90% of the tested problems and clearly outperforms the other two methods.

## Figures and Tables

**Algorithm 1:**
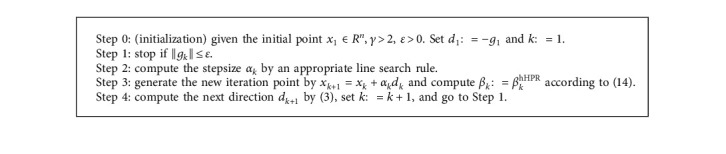
Algorithm framework.

**Table 1:** Numerical results for the three tested methods.

Problems	hHPR	DHS	DPRP
TP/Dim	Itr/Tcpu/‖*g*_*∗*_‖	Itr/Tcpu/‖*g*_*∗*_‖	Itr/Tcpu/‖*g*_*∗*_‖
bdexp/50000	2/0.157/3.51*e* − 89	2/0.136/3.51*e* − 89	2/0.138/3.51*e* − 89
bdexp/100000	2/0.223/4.58*e* − 106	2/0.223/4.58*e* − 106	2/0.220/4.58*e* − 106
bdexp/1000000	2/2.857/1.42*e* − 170	2/2.829/1.42*e* − 170	2/2.848/1.42*e* − 170
exdenschnf/50000	38/0.260/1.53*e* − 07	30/0.230/9.80*e* − 07	30/0.227/2.39*e* − 07
exdenschnf/100000	42/0.496/6.67*e* − 07	25/0.425/8.47*e* − 07	24/0.412/2.50*e* − 07
exdenschnb/5000	24/0.020/6.69*e* − 07	16/0.011/3.36*e* − 08	19/0.011/3.61*e* − 07
exdenschnb/20000	25/0.047/2.79*e* − 07	17/0.043/1.77*e* − 07	22/0.057/4.59*e* − 07
exdenschnb/100000	17/0.215/5.21*e* − 07	17/0.182/1.48*e* − 07	17/0.183/1.03*e* − 07
himmelbg/20000	2/0.029/6.91*e* − 28	2/0.022/6.91*e* − 28	2/0.021/6.91*e* − 28
himmelbg/100000	2/0.095/1.46*e* − 28	2/0.094/1.46*e* − 28	2/0.095/1.46*e* − 28
genquartic/20000	21/0.061/3.91*e* − 07	18/0.052/5.09*e* − 07	13/0.045/5.23*e* − 07
genquartic/100000	17/0.231/6.08*e* − 07	18/0.230/4.49*e* − 07	17/0.228/6.68*e* − 07
genquartic/1000000	28/3.189/1.74*e* − 07	18/2.818/5.34*e* − 07	16/2.469/4.73*e* − 07
biggsb1/200	1218/0.064/9.68*e* − 07	1607/0.103/9.61*e* − 07	1742/0.114/1.00*e* − 06
biggsb1/400	—	—	—
sine/100	95/0.013/9.33*e* − 07	29/0.003/5.63*e* − 07	27/0.003/2.45*e* − 07
sinquad/3	79/0.015/6.74*e* − 07	359/0.022/9.39*e* − 07	224/0.012/5.27*e* − 07
fletcbv3/20	101/0.014/4.42*e* − 07	110/0.006/9.01*e* − 07	147/0.008/5.29*e* − 07
fletcbv3/40	409/0.024/4.63*e* − 07	485/0.026/7.67*e* − 07	504/0.025/7.84*e* − 07
eg2/100	428/0.074/9.04*e* − 07	—	1428/0.115/7.75*e* − 07
eg2/170	1353/0.395/2.61*e* − 07	—	—
nonscomp/10000	—	—	—
nonscomp/20000	50/0.077/7.79*e* − 07	49/0.076/2.55*e* − 07	46/0.073/8.37*e* − 07
nonscomp/50000	74/0.251/6.30*e* − 07	69/0.239/6.07*e* − 07	80/0.266/8.04*e* − 07
cosine/1000	51/0.020/9.66*e* − 07	20/0.009/5.27*e* − 07	21/0.009/6.42*e* − 07
cosine/10000	51/0.090/5.31*e* − 07	33/0.072/4.32*e* − 07	21/0.057/1.04*e* − 07
dixmaana/3000	20/0.132/2.60*e* − 07	22/0.113/4.60*e* − 07	17/0.107/5.22*e* − 07
dixmaanb/3000	18/0.129/8.63*e* − 07	20/0.107/1.96*e* − 07	14/0.100/3.01*e* − 07
dixmaanc/3000	17/0.130/3.91*e* − 07	17/0.098/2.62*e* − 07	16/0.126/6.68*e* − 07
dixmaand/3000	23/0.146/2.23*e* − 07	17/0.106/4.12*e* − 07	17/0.116/3.92*e* − 07
dixmaane/3000	428/0.915/8.90*e* − 07	612/1.731/8.10*e* − 07	584/1.676/9.45*e* − 07
dixmaanf/3000	287/0.633/9.86*e* − 07	540/1.527/9.46*e* − 07	477/1.364/9.29*e* − 07
dixmaang/3000	431/0.951/7.93*e* − 07	553/1.587/9.31*e* − 07	598/1.759/8.21*e* − 07
dixmaanh/3000	538/1.292/5.87*e* − 07	960/3.049/7.59*e* − 07	918/2.984/8.35*e* − 07
dixmaanj/3000	—	—	—
dixmaank/3000	156/0.387/6.90*e* − 07	—	—
dixmaanl/3000	—	—	1199/3.390/9.95*e* − 07
dixon3dq/80	876/0.043/8.55*e* − 07	912/0.050/8.66*e* − 07	1097/0.062/9.83*e* − 07
dixon3dq/160	—	—	—
dqdrtic/10000	67/0.073/4.83*e* − 07	296/0.189/7.98*e* − 07	243/0.160/5.41*e* − 07
dqdrtic/100000	85/0.468/9.32*e* − 07	256/1.248/5.90*e* − 07	153/0.819/2.43*e* − 07
dqdrtic/1000000	70/5.007/6.70*e* − 07	265/14.500/8.11*e* − 07	273/14.436/7.49*e* − 07
dqrtic/200	27/0.014/2.68*e* − 07	24/0.008/5.68*e* − 07	26/0.009/5.17*e* − 08
dqrtic/500	33/0.021/5.05*e* − 07	34/0.023/6.73*e* − 07	29/0.020/5.84*e* − 07
edensch/1000	40/0.051/7.37*e* − 07	41/0.056/7.02*e* − 07	36/0.046/8.63*e* − 07
edensch/4000	73/0.493/8.66*e* − 07	46/0.133/3.62*e* − 07	43/0.204/8.20*e* − 07
edensch/8000	44/0.620/8.57*e* − 07	68/1.022/5.23*e* − 07	39/0.456/6.70*e* − 07
engval1/6	30/0.009/8.33*e* − 07	37/0.002/2.36*e* − 07	38/0.002/8.34*e* − 07
errinros/3	314/0.028/8.62*e* − 07	—	—
fletchcr/100	84/0.022/9.11*e* − 07	82/0.005/4.67*e* − 07	67/0.005/7.99*e* − 07
fletchcr/300	45/0.005/8.17*e* − 07	110/0.008/7.13*e* − 07	117/0.007/8.66*e* − 07
freuroth/50	263/0.047/8.81*e* − 07	788/0.107/8.23*e* − 07	664/0.054/9.91*e* − 07
genrose/5000	—	—	—
genrose/10000	177/0.152/6.78*e* − 07	499/0.433/9.48*e* − 07	776/0.659/8.02*e* − 07
genrose/20000	—	—	—

**Table 2:** Numerical results for the three tested methods (continued).

Problems	hHPR	DHS	DPRP
TP/Dim	Itr/Tcpu/‖*g*_*∗*_‖	Itr/Tcpu/‖*g*_*∗*_‖	Itr/Tcpu/‖*g*_*∗*_‖
liarwhd/5000	94/0.066/7.37*e* − 07	—	—
liarwhd/10000	138/0.186/8.02*e* − 07	—	—
liarwhd/20000	125/0.388/7.88*e* − 07	—	—
nondquar/30	534/0.072/8.10*e* − 07	760/0.085/9.19*e* − 07	—
penalty1/1000	30/0.418/2.55*e* − 07	29/0.411/2.65*e* − 07	29/0.390/9.90*e* − 07
penalty1/5000	117/39.785/2.62*e* − 07	278/103.383/4.27*e* − 07	203/72.649/1.01*e* − 07
power1/50	595/0.029/9.03*e* − 07	743/0.039/9.46*e* − 07	917/0.050/2.82*e* − 07
power1/100	1468/0.068/9.91*e* − 07	1666/0.101/6.89*e* − 07	—
quartc/100	23/0.010/6.45*e* − 08	21/0.004/3.28*e* − 07	27/0.006/1.06*e* − 07
quartc/560	32/0.023/7.15*e* − 07	29/0.020/7.08*e* − 07	33/0.022/5.70*e* − 07
tridia/100	409/0.026/6.30*e* − 07	477/0.030/3.94*e* − 07	681/0.038/9.21*e* − 07
tridia/1000	1564/0.153/9.39*e* − 07	—	—
raydan1/100	77/0.008/9.73*e* − 07	127/0.010/8.52*e* − 07	106/0.005/9.59*e* − 07
raydan1/500	210/0.015/9.14*e* − 07	281/0.023/5.80*e* − 07	268/0.022/7.43*e* − 07
raydan2/5000	12/0.027/9.72*e* − 07	12/0.021/3.54*e* − 07	12/0.023/3.54*e* − 07
raydan2/10000	12/0.051/3.75*e* − 07	13/0.052/7.31*e* − 08	13/0.050/7.31*e* − 08
raydan2/50000	16/0.239/2.43*e* − 08	16/0.217/8.05*e* − 07	17/0.272/7.70*e* − 07
diagonal1/40	61/0.010/7.58*e* − 07	81/0.005/7.52*e* − 07	63/0.004/8.67*e* − 07
diagonal2/10000	830/1.082/9.48*e* − 07	1398/2.017/9.75*e* − 07	1134/1.660/6.18*e* − 07
diagonal2/20000	1241/3.050/8.03*e* − 07	1387/4.025/9.98*e* − 07	1879/5.192/8.08*e* − 07
diagonal3/10	39/0.007/7.69*e* − 07	37/0.002/4.92*e* − 07	39/0.002/4.59*e* − 07
diagonal3/90	91/0.008/7.31*e* − 07	144/0.010/9.04*e* − 07	166/0.016/6.14*e* − 07
bv/1000	138/0.447/8.56*e* − 07	117/0.513/8.14*e* − 07	127/0.550/9.09*e* − 07
bv/2000	105/1.174/9.03*e* − 07	107/1.476/8.99*e* − 07	130/1.701/9.46*e* − 07
ie/50	16/0.051/9.24*e* − 07	11/0.039/2.20*e* − 07	15/0.044/1.83*e* − 07
ie/10	14/0.162/7.41*e* − 07	12/0.156/2.72*e* − 07	13/0.167/2.10*e* − 07
singx/100	277/0.051/3.49*e* − 07	—	—
singx/1000	565/2.397/7.35*e* − 07	—	453/1.711/5.18*e* − 07
woods/10000	177/0.175/2.74*e* − 07	589/0.492/2.25*e* − 07	814/0.631/8.15*e* − 07
band/3	19/0.012/2.13*e* − 07	15/0.002/6.71*e* − 07	18/0.002/9.14*e* − 07
bard/3	101/0.028/3.00*e* − 07	698/0.097/4.95*e* − 07	811/0.112/8.16*e* − 07
beale/2	48/0.010/6.38*e* − 07	131/0.007/2.30*e* − 07	96/0.007/8.34*e* − 07
biggs/6	—	—	—
box/3	74/0.014/5.48*e* − 07	203/0.017/1.45*e* − 07	185/0.015/5.45*e* − 07
froth/2	92/0.014/6.85*e* − 07	338/0.025/9.02*e* − 07	381/0.026/8.13*e* − 07
gauss/3	13/0.010/3.75*e* − 07	24/0.004/4.71*e* − 07	21/0.003/3.27*e* − 07
helix/3	102/0.019/4.65*e* − 07	361/0.032/9.10*e* − 07	418/0.036/9.20*e* − 07
jensam/2	39/0.009/2.79*e* − 07	148/0.009/9.70*e* − 07	149/0.009/8.40*e* − 07
kowosb/4	224/0.029/9.98*e* − 07	1029/0.075/3.25*e* − 07	797/0.056/9.64*e* − 07
lin/100	13/0.062/8.86*e* − 07	13/0.054/8.86*e* − 07	13/0.054/8.86*e* − 07
lin/500	18/0.418/9.57*e* − 07	18/0.420/9.38*e* − 07	18/0.421/9.38*e* − 07
osb2/11	734/0.113/7.42*e* − 07	1513/0.259/4.38*e* − 07	1174/0.198/7.47*e* − 07
pen1/60	78/0.025/4.74*e* − 07	390/0.056/8.20*e* − 07	668/0.079/8.95*e* − 07
pen2/100	151/0.074/7.94*e* − 07	162/0.045/5.89*e* − 07	235/0.081/8.49*e* − 07
rose/2	79/0.011/9.71*e* − 07	584/0.037/9.06*e* − 07	858/0.053/2.86*e* − 07
rosex/100	104/0.022/4.68*e* − 07	1190/0.132/7.73*e* − 07	705/0.080/8.08*e* − 07
rosex/1000	106/0.899/2.49*e* − 07	1040/6.318/8.60*e* − 07	1374/8.180/2.95*e* − 07
sing/4	213/0.023/6.12*e* − 07	—	1111/0.070/9.15*e* − 07
trid/100	80/0.021/4.41*e* − 07	112/0.022/4.22*e* − 07	128/0.024/6.31*e* − 07
trid/200	37/0.015/6.81*e* − 07	46/0.016/7.20*e* − 07	42/0.017/6.19*e* − 07
vardim/8	28/0.011/4.13*e* − 07	26/0.004/6.51*e* − 07	31/0.004/9.75*e* − 07
watson/6	1811/0.500/7.74*e* − 07	—	—
wood/4	173/0.020/6.93*e* − 07	622/0.047/5.42*e* − 07	966/0.069/9.26*e* − 07

## Data Availability

All the datasets used in this paper are available from the corresponding author upon request.
